# Determining the orientation of acetabular prosthesis in total hip arthroplasty by refering to the anatomical landmarker of acetabular notches

**DOI:** 10.1038/s41598-023-33501-8

**Published:** 2023-04-15

**Authors:** Heng Zhang, Jiansheng Zhou, Xiao Ling, Haonan Chen, Mingqiu Du, Jianning Zhao

**Affiliations:** 1 Department of Orthopedics, School of Medicine, Jinling Hospital, Nanjing University, Nanjing, Jiangsu China; 2 Laboratory of Tissue and Transplant in Anhui Province, Department of Orthopedics, The First Affiliated Hospital of Bengbu Medical College, Bengbu Medical College, Bengbu, Anhui China; 3 Clinical Medical School, Bengbu Medical College, Bengbu, China; 4 Medical Imaging School, Bengbu Medical College, Bengbu, China

**Keywords:** Anatomy, Medical research

## Abstract

The aim of this study was to explore a novel method to determine the orientation of acetabular prosthesis in total hip arthroplasty (THA) by refering to the anatomical landmarker of acetabular notches. Forty-one normal developmental hips were included in the present study. The acetabulums were reamed according to standard surgical procedures of THA on life-size 3D printing pelvis models. The inferior edge of acetabular cup were placed (1–5) mm proximal and distal to the proximal line of the anterior and posterior acetabular notches (PLAPAN) respectively to determine cup inclination. The inferior edge of acetabular cup were placed (1–5) mm pronating and supinating around the proximal point of acetabular posterior notch (PPAPN) respectively to determine cup anteversion. The pelvis plain radiographs were took and the inclination and anteversion of the acetabular cup at 22 positions were calculated. In the normal developmental hip, the mean inclination of acetabular prothesis were (35.10 ± 3.22)° and (45.90 ± 2.68)° when the inferior edge of the acetabular cup was 3 mm proximal and 1 mm distal to the PLAPAN. The optimal cup inclination could be obtained when the inferior edge of the acetabular cup was 1 mm proximal to the PLAPAN (the mean inclination was (40.71 ± 2.80)°). The mean anteversion of acetabular prothesis were (10.67 ± 4.55)° and (20.86 ± 4.44)° when the inferior edge of the acetabular cup was 1 mm pronating and 1 mm supinating around the PPAPN. The optimal cup anteversion could be obtained when the inferior edge of the acetabular cup was parallel to the PLAPAN (the mean anteversion was (18.00 ± 1.64)°). The inclination and anteversion of acetabular prosthesis could be determined by refering the anatomical landmarks of acetabular notches, which could help orthopedists to install the acetabular prosthesis quickly and safely in THA.

## Introduction

The installation position of the acetabular prosthesis in total hip arthroplasty (THA) is paramount, which affect the matching relationship with the artificial femoral head in the process of motion as well as the recovery of hip joint function^1^. Poor position of acetabular prosthesis can easily lead to dislocation, impingement, wear, and loosening, which reduce life expectancy of the prosthesis and bring in hazard to patients^2,3^.

Three parameters should be considered for precise installation of acetabular prosthesis in THA: hip center of rotation (HCR), inclination and anteversion. The change of HCR in THA could result in lower limb length discrepancy (LLD), gait changes, increased wear and loosening of the prosthesis. The restoration of HCR help to balance the hip joint stress and recover soft tissue tension, thus prolonging the service life of the prosthesis^4^. At present, many methods have been used to locate the HCR, however, most of which were based on two-dimensional X-ray measurement. These methods are of limited guidance value in the operation. With respect to the anteversion of the acetabular cup, many scholars believed that satisfactory anteversion could be obtain when the inferior edge of the acetabular cup was placed parallel to the transverse acetabular ligament^5–7^. However, not all of the transverse acetabular ligament could be separated in the operation, especially Beverland grade four^8^. Epstain et al.^9^ found that only 47% of the acetabular transverse ligament could be separated in 64 cases of primary total hip arthroplasty. Griffin et al.^10^ performed CT scans for 320 hip joints, only 218 of which had significant transverse acetabular ligament. There were few studies on how to determine the inclination of acetabular cup reference to anatomical landmarkers. The anterior and posterior acetabular notches were located at the inferior edge of the acetabulum. The acetabular fossa was located at the bottom of the acetabulum without articular cartilage coverage. Both the acetabular notches and acetabular fossa have relative constant anatomical position and features. Therefore, we speculated the acetabular notches and acetabular fossa were proper anatomical landmarkers of acetabular prosthesis localization. In previous studies, we located the acetabular center in THA using the acetabular fossa and acetabular notches as the landmarks and restored the rotation center exactly, even in developmental dysplasia of the hip (DDH) and revision THA cases^11–13^.

Therefore, the purposes of this study were (1) to determine the anteversion and inclination of acetabular prosthesis using acetabular notche as anatomical landmarkers and (2) determine the intra-operative positional safe zone range of the acetabular prosthesis’ anteversion and inclination.

## Materials and methods

### Study design

This study was approved by the institutional review board of the First Affiliated Hospital of Bengbu Medical College, and informed consent was obtained from all involved patients. All methods were carried out in accordance with relevant guidelines and regulations.

### Study subjects

This study involved 25 patients (41 hips) who were admitted to our institution from September 2018 to October 2020. The inclusion criteria were: (1) age greater than or equal to 18 years old; (2) hip fractures with normal acetabulum; osteonecrosis of femoral head (Ficat stage I to IV); one side of pelvis and acetabular fractures with normal contralateral side. The exclusion criteria were: (1) DDH (Crowe type I to IV, Lateral center edge angle < 20°, Tönnis angle > 10°) and borderline DDH (Lateral center edge angle: 20°–25°); (2) severe acetabular deformation due to end-stage hip diseases; (3) plevic and acetabular tumors.

### Preparation of 3D printing pelvis models

Pelvic three dimensional (3D) CT scans (120 kV; 200 mAs; 0.625 mm thick slices, 0.625 mm interlamellar spacing) of all 25 patients were performed (Brilliance 64-slice spiral CT, Philips Investment Co. LTD, Netherlands) and the data was imported into Mimics 10.01 software (Materialise, Belgium). 3D pelvis models were reconstructed and exported in STL format. The Ultimaker Cura 3.0 software (Ultimaker, Netherlands) was used to slice up the model. Life-size pelvic models were printed by a 3D printing machine (Arigin 3DM400, Shanghai Arigin Medical Technology Co. LTD, China) using polylactic acid (PLA) material (220 V; 15 A; 0.25 mm layer sickness, 1.75 mm printing material diameter, 80 mm/s printing speed; 200°nozzle temperature, 50° hot bed temperature).

**Figure 1:**
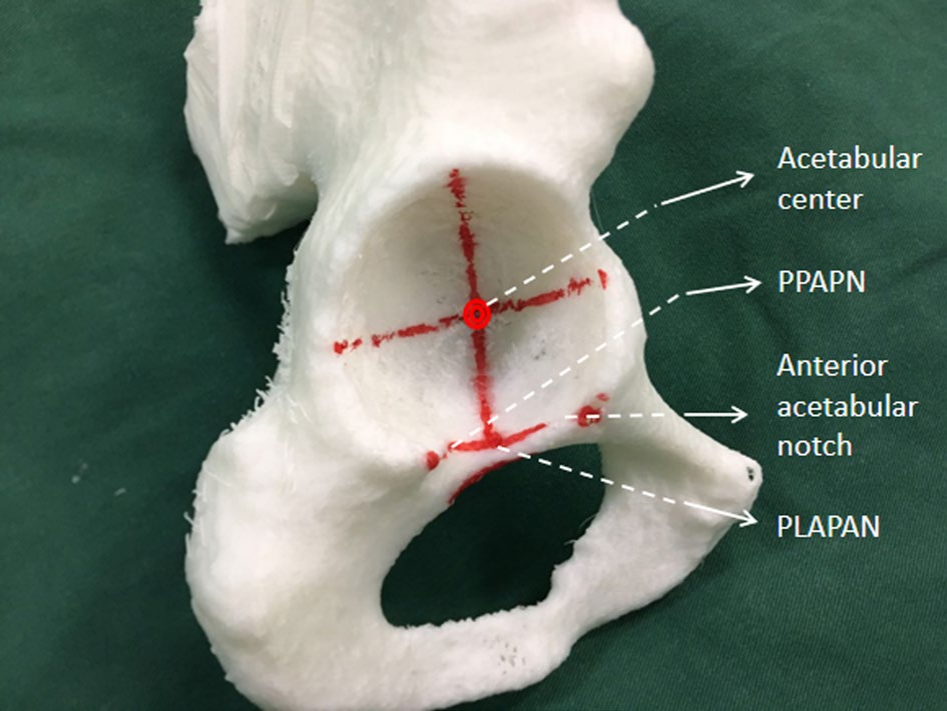
Acetabulum model and anatomical landmarks (*PPAPN* proximal point of acetabular posterior notch, *PLAPAN* proximal line of the anterior and posterior acetabular notches).

The acetabulums were reamed according to standard surgical procedures of THA on the pelvis models. The acetabular center was located according to the method proposed by Heng Zhang et al.^13^, that is, the acetabular center was located at 25–31 mm (mean 28.7 mm, depended on the size of the acetabulum) above the intersection point of the perpendicular bisector and acetabular anterior and posterior notches’ line. During the process of acetabulum reaming, the anteversion was controlled at 15°, while the inclination was 40°, and the depth was the bottom of acetabular fossa. Concentric Reaming was performed from small to large, aiming at the acetabular center. The final cup size was determined by the criterion that the anterior and posterior wall had sufficient clamping force to obtain good initial stability of the test cup. The anterior and posterior acetabular notches and their proximal lines were marked (Fig. 1).

### The orientation of acetabular cup’s inclination and anteversion by refering to the acetabular notches

**Figure 2:**
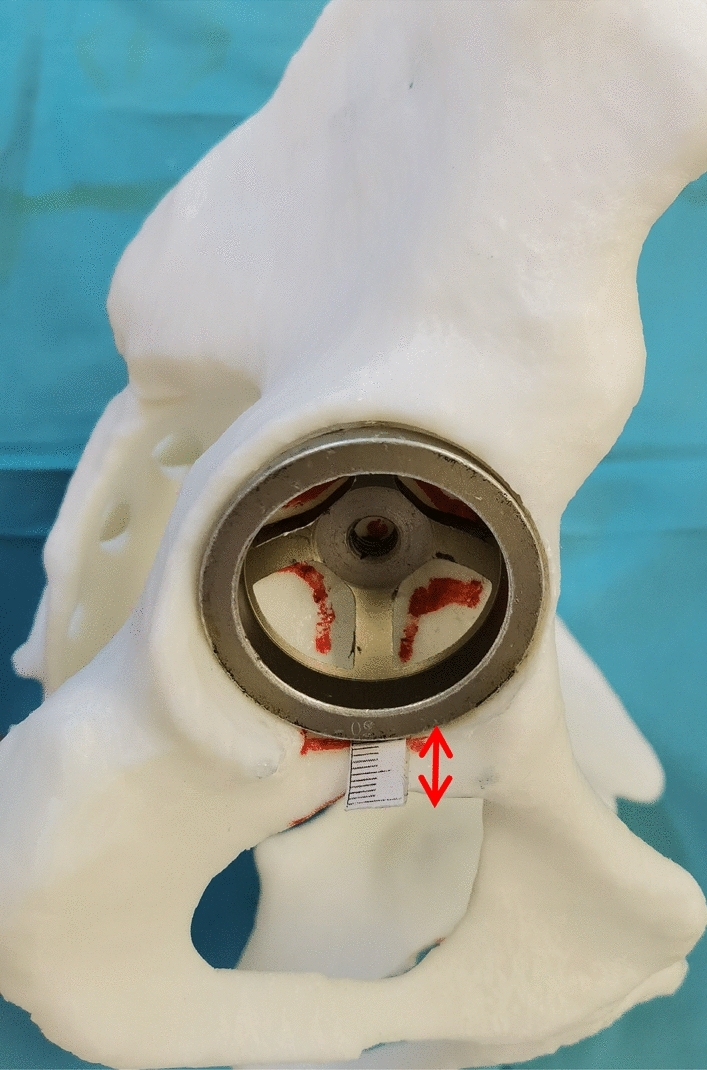
The position relationship between the inferior edge of acetabular cup and PLAPAN to determine cup inclination.

**Figure 3:**
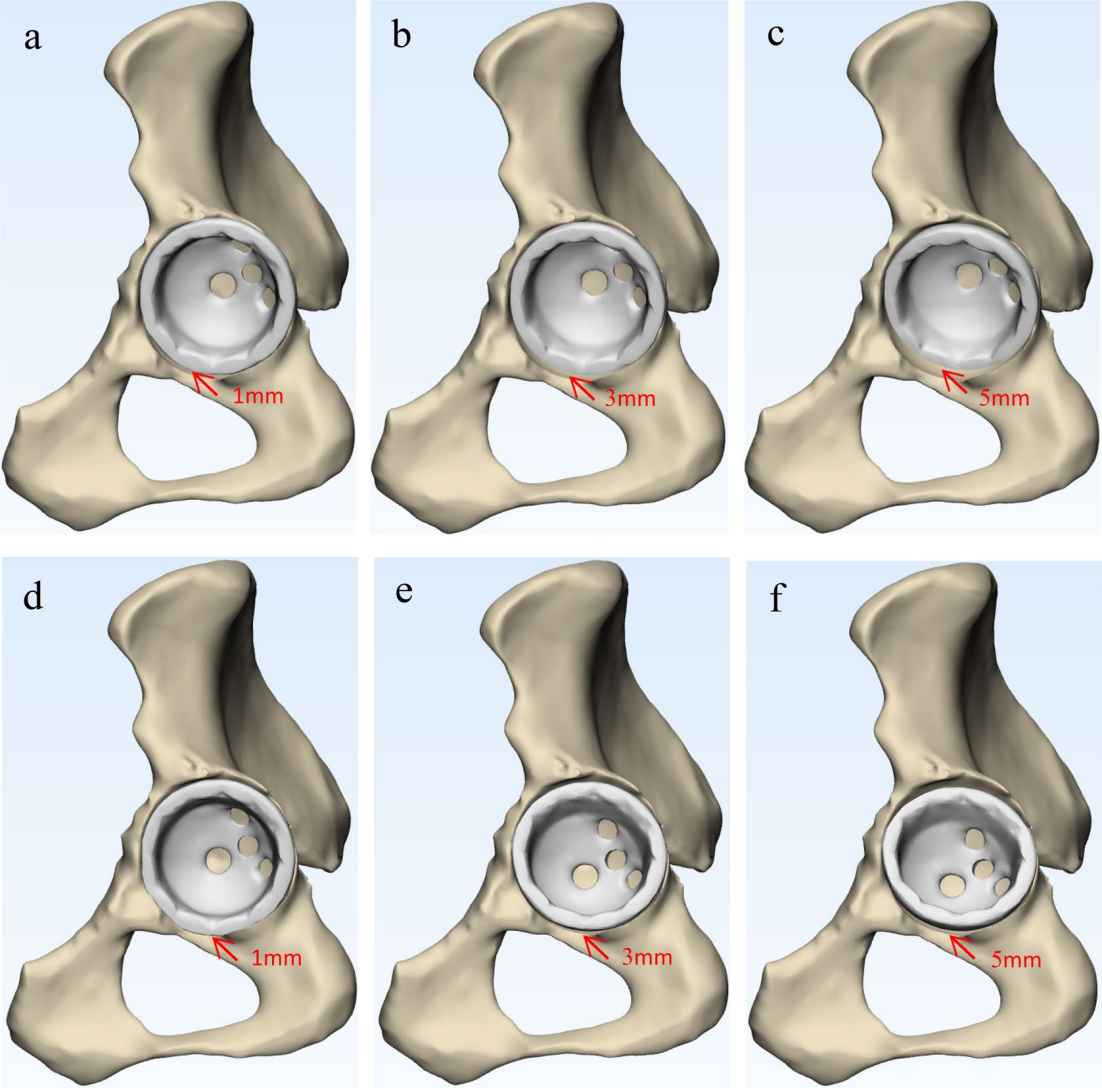
The position relationship between the inferior edge of acetabular cup and the proximal line of the anterior and posterior acetabular notches to determine cup inclination. (**a**) 1 mm proximal to the PLAPAN; (**b**) 3 mm proximal to the PLAPAN; (**c**) 5 mm proximal to the PLAPAN; (**d**) 1 mm distal to the PLAPAN; (**e**) 3 mm distal to the PLAPAN; (**f**) 5 mm distal to the PLAPAN.

A self-made paper ruler was pasted at the middle of the proximal line of the anterior and posterior acetabular notches (PLAPAN). Double-sided tape was wrapped around the acetabular cup. The up-down, left–right tracks of the acetabular cup movement were marked on the model (Fig. 2). Kept the inferior edge of the acetabular cup be parallel to the PLAPAN during the acetabular cup movement in order to maintain the anteversion be unchanged. The inferior edge of acetabular cup were placed (1–5) mm proximal and distal to the PLAPAN respectively to determine cup inclination (Fig. 3).

**Figure 4:**
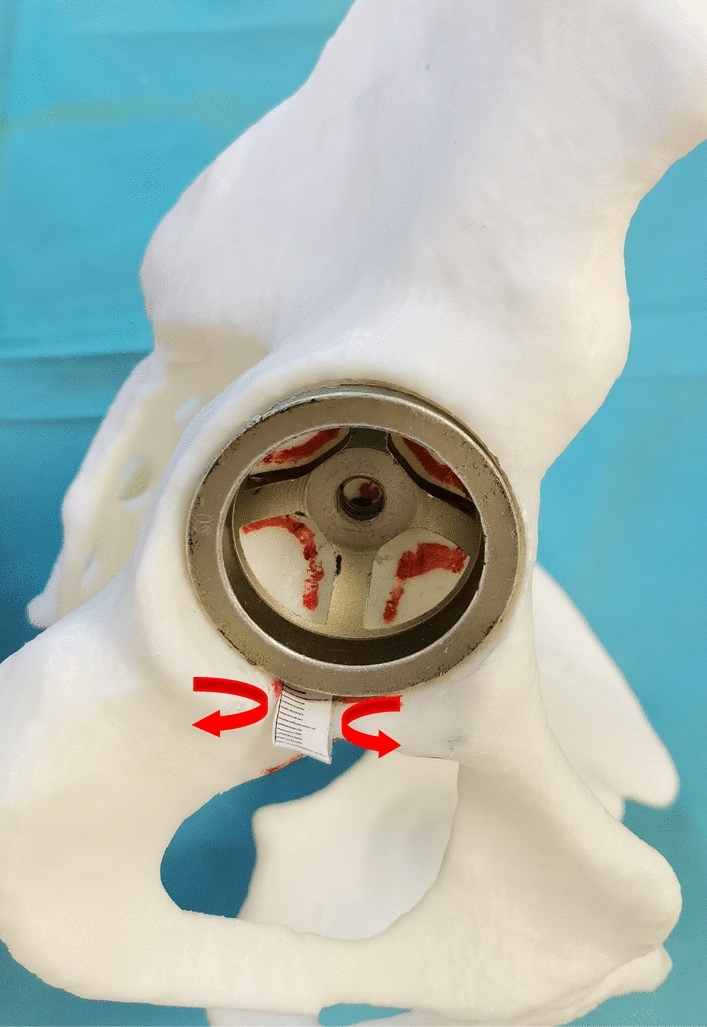
The position relationship between the inferior edge of acetabular cup and PPAPN to determine cup anteversion.

**Figure 5:**
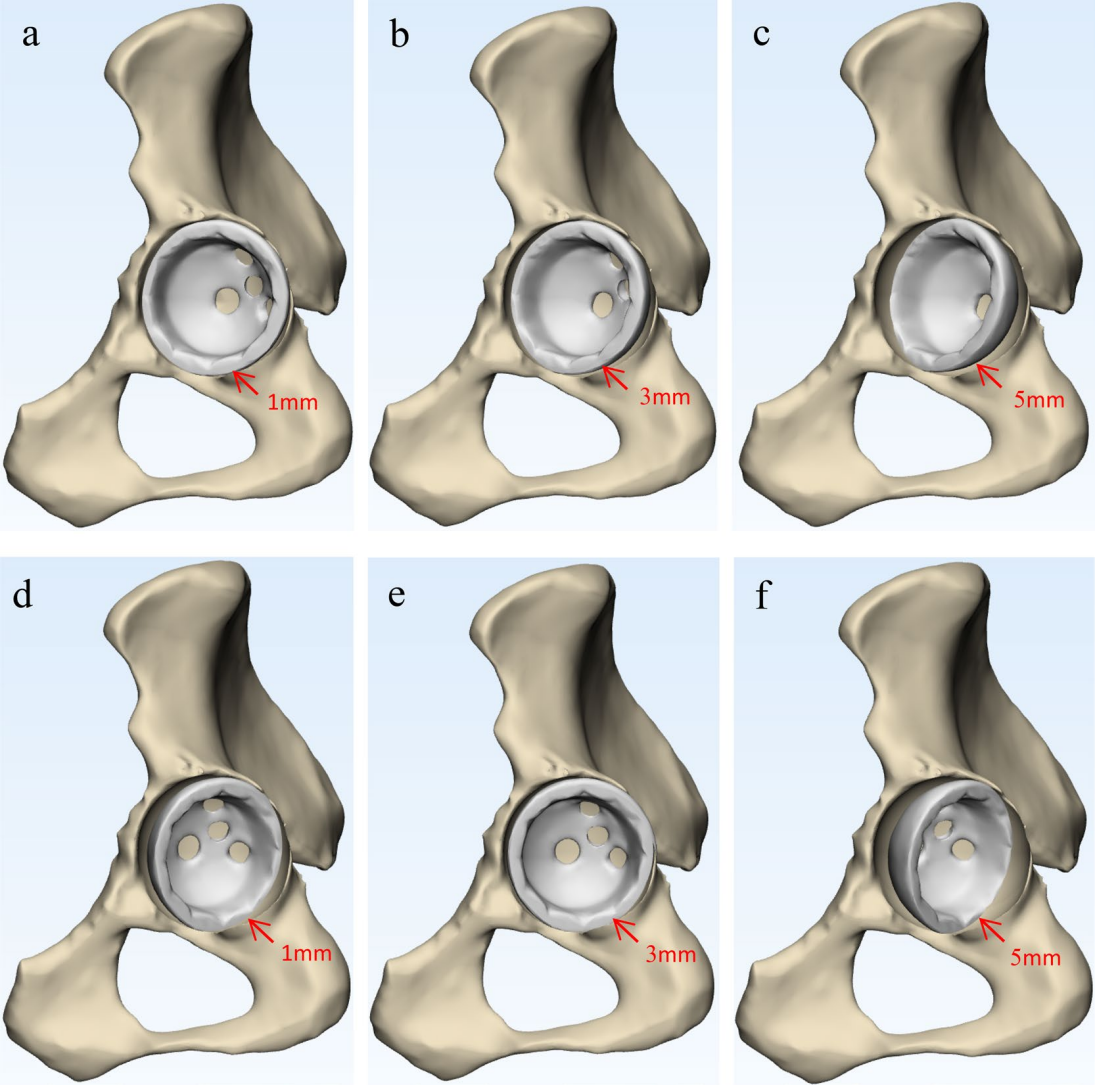
The position relationship between the inferior edge of acetabular cup and the proximal line of the anterior and posterior acetabular notches to determine cup anteversion. (**a**) 1 mm pronating around the PPAPN; (**b**) 3 mm pronating around the PPAPN; (**c**) 5 mm pronating around the PPAPN; (**d**) 1 mm supinating around the PPAPN; (**e**) 3 mm supinating around the PPAPN; (**f**) 5 mm supinating around the PPAPN.

A self-made paper ruler was pasted at the proximal point of the posterior acetabulum notch (PPAPN) (Fig. 4). Kept the midpoint of the inferior edge of the acetabular cup overlap the midpoint of the PLAPAN in order to maintain the inclination be unchanged during the acetabular cup movement. The inferior edge of acetabular cup were placed (1–5) mm pronating and supinating around the PPAPN respectively to determine cup anteversion (Fig. 5).

Standard plain films of the pelvis model were taken by C-arm X-ray machine(Siemens AG, Germany).The standard of pelvic plain film are as follow: ① the tip of the coccygeal bone points to the midpoint of the symphysis pubis; ② the line of bilateral tear drops’ lower margin is perpendicular to the central axis of lumbosacral vertebra; ③ the distance between the middle point of the superior margin of the pubic symphysis and the sacrococcygeal joint was (1–4) cm in males and (4–6) cm in females; ④ the size of bilateral obturators are basically equal. The images were imported into GeoGebraGeometry software (V6.0.574.0, America). The inclination was calculated by the classical method, that is, the angle between the elliptic (formed by the acetabular cup edge) long axis and the line of bilateral teardrops. The Liaw method was used to calculate the anteversion, that is, the anteversion was equal to arcsin (A/B). A and B were the short axis and the long axis of the ellipse formed by the acetabular cup edge respectively. Two observers measured the inclination and anteversion of the acetabular cup at 11 positions three times, averaged the results to obtain the data and repeated after three weeks on the same computer.

### Statistical analysis

All statistical analyses were performed by using SPSS software for Windows (version 19.0; SPSS, Chicago, IL, USA). Continuous variables were presented as means and ranges.Analysis of Variance (ANOVA) was used to compare the differences in genders, left–right sides of the acetabular cup’s inclination and anteversion at 11 positions. Intraclass correlation coefficient (ICC) was used to assess the intra-observer and inter-observe reliability of measurements of the acetabular cup’s inclination and anteversion. A p value < 0.05 was considered statistically significant.

## Results

### ICC of observers

The ICC values of intra-observer and inter-observe were both greater than 0.9, suggesting good reliability of the measurement results. However, the intra-observer and inter-observer’s ICC values of anteversion were lower than that of inclination, indicating that the measurement method of the anteversion had certain subjectivity, which existed in the measurement of the long axis and the short axis of the ellipse formed by the edge of the acetabular cup (Table 1).

**Table 1:** The ICC value of observers’ measurement data of acetabular cup’s anteversion and inclination.

Observer	Inclination	Anteversion
Observer 1	0.98	0.93
Observer 2	0.97	0.92
Interobserver reliability	0.95	0.90

### The statistical differences in genders, left–right sides of the acetabular cup’s inclination and anteversion at 11 positions

There was no statistically significant differences in genders of the 11 positions of the acetabular cup’s inclination and anteversion (p > 0.05). No statistically significant variation was observed in left–right sides of the acetabular cup’s inclination and anteversion at 11 positions (p > 0.05) (Tables 2, 3, 4, 5).

**Table 2:** The comparison of cup’s inclination in different genders of normal developmental hips (x ± s).

	N	0 mm	− 1 mm	− 2 mm	− 3 mm	− 4 mm	− 5 mm	1 mm	2 mm	3 mm	4 mm	5 mm
Genders												
Male	21	43.24 ± 2.55	40.38 ± 2.71	37.57 ± 2.84	34.67 ± 2.48	32.10 ± 2.57	29.52 ± 2.94	45.48 ± 2.42	48.57 ± 3.09	51.29 ± 3.49	54.57 ± 4.60	57.04 ± 4.98
Female	20	43.65 ± 2.52	41.05 ± 2.93	38.55 ± 3.49	35.55 ± 3.86	32.5 ± 4.35	29.85 ± 4.32	46.35 ± 2.92	49.85 ± 4.78	52.95 ± 5.40	56.40 ± 6.31	59.35 ± 6.74
F		0.271	0.577	0.976	0.138	0.595	0.081	1.090	1.044	1.385	1.132	1.558
*P*		0.606	0.452	0.329	0.712	0.445	0.778	0.303	0.313	0.246	0.294	0.219

**Table 3:** The comparison of cup’s inclination in different sides of normal developmental hips (x ± s).

	N	0 mm	− 1 mm	− 2 mm	− 3 mm	− 4 mm	− 5 mm	1 mm	2 mm	3 mm	4 mm	5 mm
Sides												
Left	21	43.76 ± 2.28	40.86 ± 2.63	38.29 ± 2.72	35.43 ± 3.06	32.85 ± 3.54	30.19 ± 3.08	46.09 ± 2.76	49.24 ± 3.94	52.23 ± 4.68	55.90 ± 6.03	58.57 ± 6.25
Right	20	43.10 ± 2.75	40.55 ± 3.03	37.80 ± 3.64	34.75 ± 3.42	32.15 ± 3.57	29.15 ± 4.16	45.70 ± 2.66	49.15 ± 4.18	51.95 ± 4.52	55.00 ± 5.02	57.75 ± 5.73
F		0.707	0.120	0.236	0.450	0.405	0.835	0.218	0.005	0.040	0.271	0.192
*P*		0.406	0.731	0.630	0.506	0.528	0.366	0.643	0.945	0.842	0.605	0.664

**Table 4:** The comparison of cup’s anteversion in different genders of normal developmental hips (x ± s).

	N	0 mm	− 1 mm	− 2 mm	− 3 mm	− 4 mm	− 5 mm	1 mm	2 mm	3 mm	4 mm	5 mm
Genders												
Male	21	17.90 ± 1.67	13.33 ± 3.50	8.19 ± 3.64	4.14 ± 4.43	− 0.24 ± 6.39	− 3.71 ± 7.20	21.90 ± 2.51	28.29 ± 4.70	34.52 ± 6.71	39.90 ± 7.64	45.10 ± 8.67
Female	20	18.15 ± 1.60	13.35 ± 2.16	8.25 ± 5.27	3.90 ± 6.73	− 0.25 ± 9.05	− 4.45 ± 10.12	21.85 ± 2.58	27.50 ± 4.63	33.60 ± 6.89	39.70 ± 7.50	46.60 ± 9.25
F		0.230	0.000	0.002	0.019	0.000	0.072	0.005	0.290	0.189	0.007	0.289
*P*		0.634	0.896	0.967	0.892	0.996	0.789	0.945	0.593	0.666	0.932	0.594

**Table 5:** The comparison of cup’s anteversion in different sides of normal developmental hips (x ± s).

	N	0 mm	− 1 mm	− 2 mm	− 3 mm	− 4 mm	− 5 mm	1 mm	2 mm	3 mm	4 mm	5 mm
Sides												
Left	21	18.24 ± 1.48	13.57 ± 2.79	8.24 ± 5.49	4.00 ± 6.68	− 0.38 ± 9.29	− 4.29 ± 10.48	22.29 ± 2.31	29.14 ± 4.65	35.67 ± 7.43	40.86 ± 8.00	47.05 ± 9.79
Right	20	17.80 ± 1.77	13.10 ± 3.04	8.20 ± 3.16	4.05 ± 4.34	− 0.10 ± 5.82	− 3.85 ± 6.45	21.45 ± 2.70	26.60 ± 4.33	32.40 ± 5.60	38.70 ± 6.95	44.55 ± 7.86
F		0.744	0.268	0.001	0.001	0.013	0.025	1.138	3.273	2.509	0.845	0.807
*P*		0.394	0.607	0.979	0.978	0.909	0.874	0.293	0.078	0.121	0.363	0.375

### The orientation of acetabular cup’s inclination and anteversion

**Figure 6:**
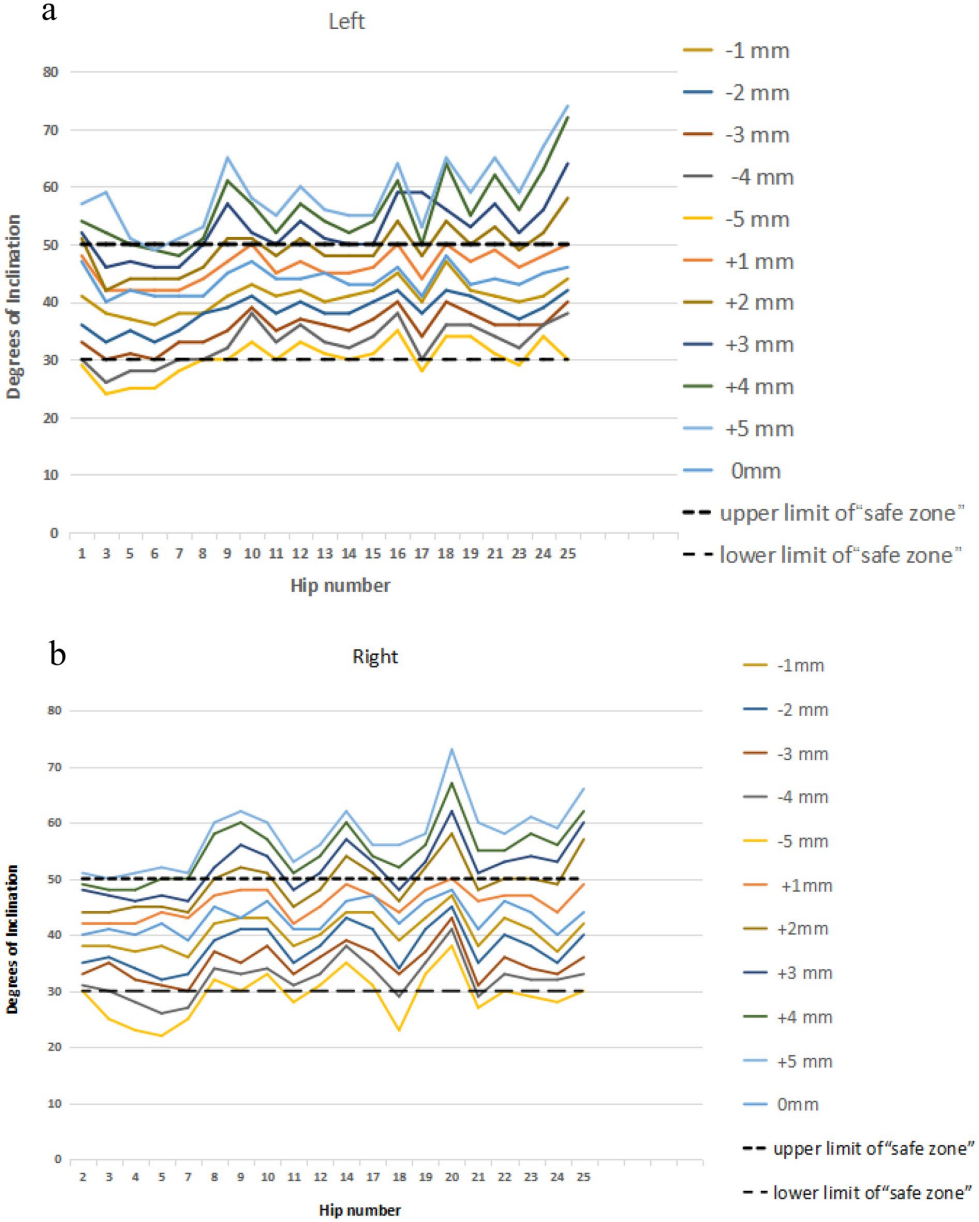
Cup’s inclination of 11 different cup positions in relation to Lewinnek’s safe zone for inclination ((**a**) left hips; (**b**) right hips).

**Figure 7:**
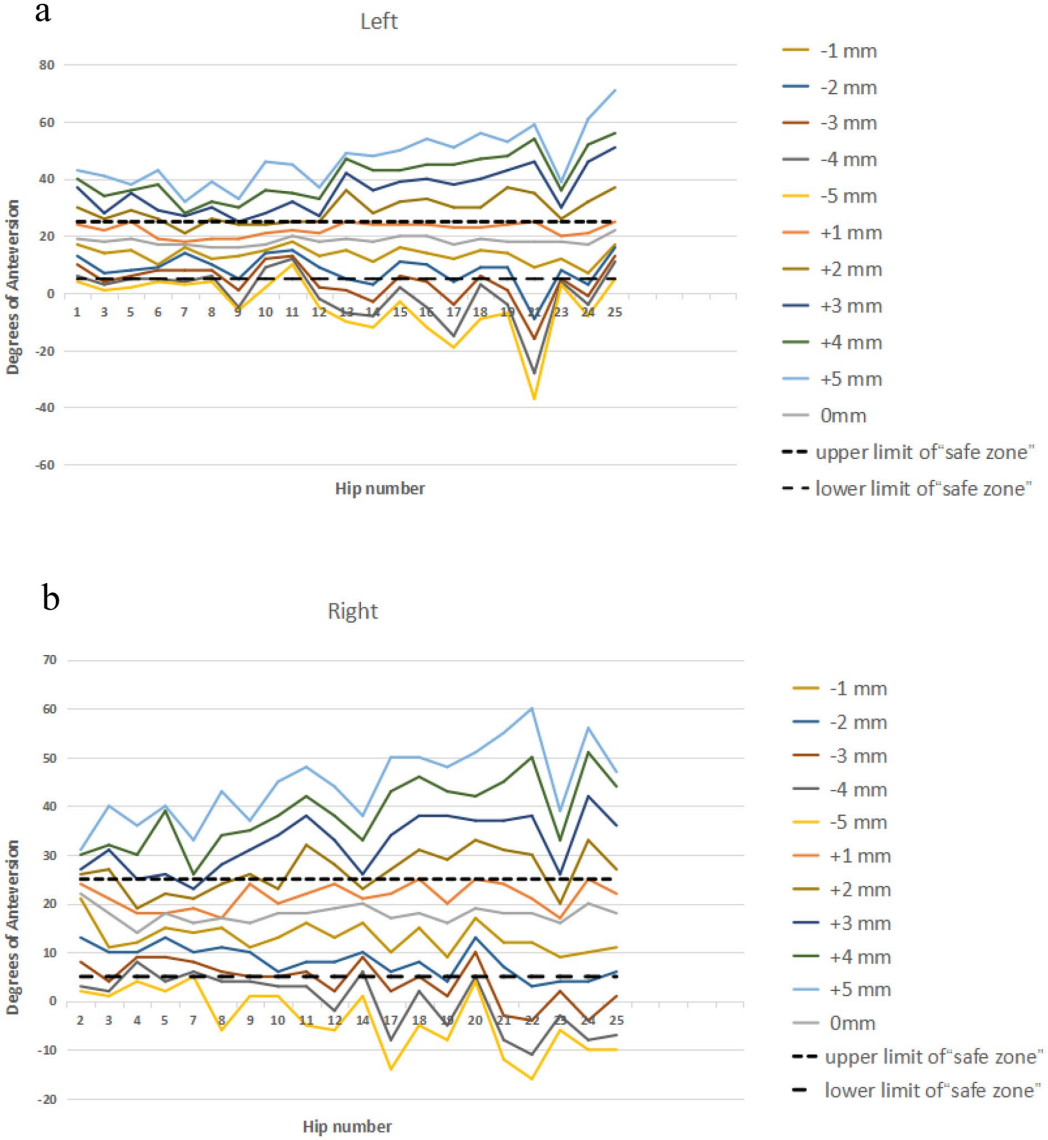
Cup’s anteversion of 11 different cup positions in relation to Lewinnek’s safe zone for anteversion ((**a**) left hips; (**b**) right hips).

**Figure 8:**
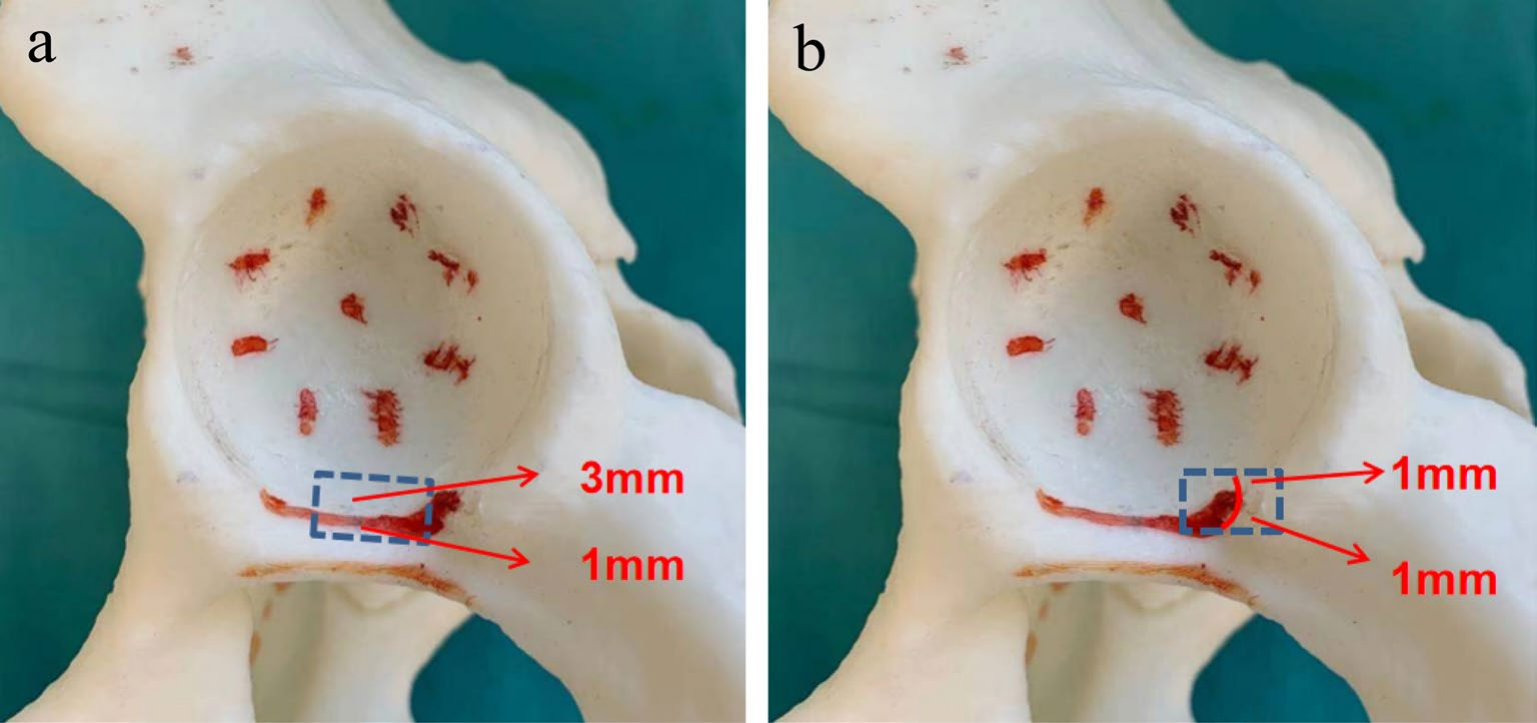
(**a**) Intraoperative positional safe zone range of cup’s inclination; (**b**) intraoperative positional safe zone range of cup’s anteversion.

The mean inclination of acetabular prothesis were (35.10 ± 3.22)°–(45.90 ± 2.68)° when the inferior edge of the acetabular cup was 3 mm proximal—1 mm distal to the PLAPAN (Figs. 6, 8a). The optimal cup inclination could be obtained when the inferior edge of the acetabular cup was 1 mm proximal to the PLAPAN (the average inclination was (40.71 ± 2.80)°) (Fig. 6). The mean anteversion of acetabular prothesis were (10.67 ± 4.55)°–(20.86 ± 4.44)° when the inferior edge of the acetabular cup was 1 mm pronating—1 mm supinating around the PPAPN (Figs. 7, 8b). The optimal cup anteversion could be obtained when the inferior edge of the acetabular cup was parallel to the PLAPAN (the average anteversion was (18.00 ± 1.64)°) (Fig. 7).

## Discussion

Three parameters should be considered for precise orientation of acetabular prosthesis: rotation center, inclination and anteversion. However, at present, there is still lack of a reliable, operable and repeatable method to guide surgeons to install the acetabular prostheses accurately in THA.

In this study, the Lewinnek radiographic “safe zone”^14^ was transformed into intraoperative positional “safe zone”. Based on the data analysis, the value of orientating the inclination and anteversion of the acetabular cup using the acetabular notches as the reference anatomical landmark was elucidated. The intraoperative positiongal “safe zone” range of the inclination and anteversion were also discussed. It was the further research on the basis of the previous study of locating the hip rotation center using the anterior and posterior acetabular notches and acetabular fossa as reference anatomical landmarks.

Small inclination of acetabular prosthesis could result in limited motion of hip flexion and abduction. During the process of hip abduction, the greater trochanter and the outer edge of acetabular cup are prone to impinge. However, large inclination of acetabular prosthesis contribute to inadequate coverage of the femoral head and limited motion of hip adduction and rotation, as well as increase the risk of upward dislocation and the wear rate of highly cross-linked polyethylene^15^. Proper inclination of acetabular prosthesis could avoid hip impingement, dislocation and maintain good range of motion and joint stability^16^. Sotereanos et al.^17^ established a reference plane to locate the anteversion and inclination of the acetabular prosthesis by using the reference of three bony anatomical markers of the upper ischium, the superior pubis ramus and the upper acetabular margin. This method was used in 617 cases of THA, which obtained 44.4° of the average inclination and 13.2° of the average anteversion. Li^18^ marked the central axis of the acetabular fossa on 16 normal adult pelvic specimens, and observed the relationship between the intersection point of the central axis and the fossa apex as well the osseous edge of the acetabulum and their projection points on standard pelvis X-ray films, so as to guide the location of the acetabular cup’s inclination. However, in clinical practice, it is difficult to clearly expose the acetabular margin due to hyperplasia and deformation. The reference value of these methods are limited. Hiddema et al.^19^ measured the inclination of the acetabular cup in three positions with the inferior edge of cup flush with, 5 mm proximal to, and 5 mm distal to the transverse acetabular ligament (TAL), which obtained median inclination 44°, 30° and 64° respectively. However, not all transverse acetabular ligaments can be dissected clearly in THA^10,20^.

The anteversion is paramount with respect to the orientation of acetabular prothesis^21^. Hassan et al.^22^ emphasized the requirement for an accurate intraoperative method to determine the anteversion, due to the mistake of 21 of every 50 installed acetabular cups outside the Lewinnek “safe zone”, even for experienced orthopedic surgeons. Zhang et al.^23^ reported that the femoral anteversion of DDH increased and the combined anteversion should be taken into consideration in THA so as to avoid dislocation and acquire well-function of hip joint.

The transverse acetabular ligament is the reference anatomical landmark most commonly used to guide the orientation of the acetabular cup’s anteversion. Idrissi et al.^7^ used the transverse acetabular ligament as the anatomical landmark to guide the placement of the acetabular cup and obtained a mean 16.9° of the acetabular cup’s anteversion, suggested that the TAL was as an important anatomical landmark to assist the orientation of the acetabular cup in THA. Archbold et al.^20^ reported a 0.6% dislocation rate in 1000 patients when made the lower margin of the acetabular prosthesis be parallel to the TAL to determine the anteversion. However, as mentioned above, the TAL exists a certain rate of loss and individual differences. Viste et al.^24^ tried to prove whether the TAL could be used as an individual anatomical landmark to guide the orientation of the acetabular cup in a cadaver study, while the results showed that the TAL as a reference anatomical landmark to guide the location of the acetabular cup remained to be verified.

Compared with the TAL, the acetabular fossa and acetabular notches are bony structures, which are more constant. Zhang et al.^12^ found that even in hip revision cases, the remnant of acetabular fossa and acetabular notches could still be found. This study applied the acetabular fossa and acetabular notches as reference anatomical landmarks to guide the orientation of the inclination and anteversion of acetabular cup, which has never be reported to our knowledge. In DDH cases, the morphology of the acetabular fossa and acetabular notches could be restored by removing superficial osteophytes. The proximal lines of anterior and posterior acetabular notches are basically parallel to the upper edge of the TAL, which is easy to identify in THA. Therefore, this method is operable and reliable in THA to some extent.

There are several limitations to our study. The limitations of this study are: First, the sample size was too small to cover all the acetabular morphology observed in clinical practice; Second, the elastic modulus of 3D printed PLA material was different from that of bone, the clamping force of which was weak, result in the final acetabular cup size of this experiment was smaller than that of the clinical practice. Third, It was difficult to achieve unified standard in pushing the acetabular cup and taking pictures, causing errors to some extent. Fourth, the divergence regarding to radiological position of pelvis model between the clinical setting and the setting in our experiment may affect the measurement results of the cup’s anteversion and the inclination a little. Last, plain film is the most common radiological examination due to low cost and dose radiation in clinical practice. However, three-dimensional computed tomography is the best way to evaluate the acetabular prosthesis position. In this study, we selected pelvis plain film as the radiological evaluation examination of acetabular cup position, which affected the accuracy of the measurement results of acetabular cup’s anteversion and inclination to some extent.

Although the Lewinnek radiographic “safe zone” has been widely used to evaluate the position of acetabular prosthesis, while this concept has little value of guiding the intraoperative orientation of acetabular cup. This study applied the relative constant anatomical landmarks of anterior and posterior acetabular notches to orientate the inclination and anteversion of the acetabular prosthesis innovatively and pointed out the acetabular cup’s position of the optimal inclination and anteversion in THA. This study proposed the intraoperative positional safe zone range of inclination and anteversion of acetabular prothesis for the first time, which could help orthopedists to orientate the inclination and anteversion of the acetabular prosthesis quickly and safely.

## Supplementary Information

Supplementary Information.

## Data Availability

All data generated or analysed during this study are included in this published article and its Supplementary Information files.
