# Supplementary material for: Determining the orientation of acetabular prosthesis in total hip arthroplasty by refering to the anatomical landmarker of acetabular notches
Source: Sci Rep. 2023 Apr 15;13:6185. doi: 10.1038/s41598-023-33501-8 (PMC10105719; doi:10.1038/s41598-023-33501-8)
Supplement: Supplementary file 1 — Supplementary Information. [file 41598_2023_33501_MOESM1_ESM.doc]

**Acetabular cup’s inclination at 11 positions**

| Hip number | Inclination（°） | | | | | | | | | | | Anteversion（°） |
| --- | --- | --- | --- | --- | --- | --- | --- | --- | --- | --- | --- | --- |
|  | 0mm | -1 | -2 | -3 | -4 | -5 | +1 | +2 | +3 | +4 | +5 |  |
| 1 L | 47 | 41 | 36 | 33 | 30 | 29 | 48 | 51 | 52 | 54 | 57 | 19 |
| 2 R | 40 | 38 | 35 | 33 | 31 | 30 | 42 | 44 | 48 | 49 | 51 | 22 |
| 3 L | 40 | 38 | 33 | 30 | 26 | 24 | 42 | 42 | 46 | 52 | 59 | 18 |
| R | 41 | 38 | 36 | 35 | 30 | 25 | 42 | 44 | 47 | 48 | 50 | 18 |
| 4 R | 40 | 37 | 34 | 30 | 28 | 23 | 42 | 45 | 46 | 48 | 51 | 14 |
| 5 L | 42 | 37 | 35 | 31 | 28 | 25 | 42 | 44 | 47 | 50 | 51 | 19 |
| R | 42 | 38 | 32 | 29 | 26 | 22 | 44 | 45 | 47 | 50 | 52 | 18 |
| 6 L | 41 | 36 | 33 | 30 | 28 | 25 | 42 | 44 | 46 | 49 | 49 | 17 |
| 7 L | 41 | 38 | 35 | 33 | 30 | 28 | 42 | 44 | 46 | 48 | 51 | 17 |
| R | 39 | 36 | 33 | 30 | 27 | 25 | 43 | 44 | 46 | 50 | 51 | 16 |
| 8 L | 41 | 38 | 38 | 33 | 30 | 30 | 44 | 46 | 50 | 51 | 53 | 16 |
| R | 45 | 42 | 39 | 37 | 34 | 32 | 47 | 50 | 52 | 58 | 60 | 16 |
| 9 L | 45 | 41 | 39 | 35 | 32 | 30 | 47 | 51 | 57 | 61 | 65 | 16 |
| R | 43 | 43 | 41 | 35 | 33 | 30 | 48 | 52 | 56 | 60 | 62 | 16 |
| 10 L | 47 | 43 | 41 | 39 | 38 | 33 | 50 | 51 | 52 | 57 | 58 | 17 |
| R | 46 | 43 | 41 | 38 | 34 | 33 | 48 | 51 | 54 | 57 | 60 | 18 |
| 11 L | 44 | 41 | 38 | 35 | 33 | 30 | 45 | 48 | 50 | 52 | 55 | 20 |
| R | 41 | 38 | 35 | 33 | 31 | 28 | 42 | 45 | 48 | 51 | 53 | 18 |
| 12 L | 44 | 42 | 40 | 37 | 36 | 33 | 47 | 51 | 54 | 57 | 60 | 18 |
| R | 41 | 40 | 38 | 36 | 33 | 31 | 45 | 48 | 51 | 54 | 56 | 19 |
| 13 L | 45 | 40 | 38 | 36 | 33 | 31 | 45 | 48 | 51 | 54 | 56 | 19 |
| 14 L | 43 | 41 | 38 | 35 | 32 | 30 | 45 | 48 | 50 | 52 | 55 | 18 |
| R | 46 | 44 | 43 | 39 | 38 | 35 | 49 | 54 | 57 | 60 | 62 | 20 |
| 15 L | 43 | 42 | 40 | 37 | 34 | 31 | 46 | 48 | 50 | 54 | 55 | 20 |
| 16 L | 46 | 45 | 42 | 40 | 38 | 35 | 49 | 54 | 59 | 61 | 64 | 20 |
| 17 L | 41 | 40 | 38 | 34 | 30 | 28 | 44 | 48 | 49 | 50 | 53 | 17 |
| R | 47 | 44 | 41 | 37 | 34 | 31 | 47 | 51 | 53 | 54 | 56 | 17 |
| 18 L | 48 | 47 | 42 | 40 | 36 | 34 | 50 | 54 | 56 | 64 | 65 | 19 |
| R | 42 | 39 | 34 | 33 | 29 | 23 | 44 | 46 | 48 | 52 | 56 | 18 |
| 19 L | 43 | 42 | 41 | 38 | 36 | 34 | 47 | 50 | 53 | 55 | 59 | 18 |
| R | 46 | 43 | 41 | 37 | 35 | 33 | 48 | 52 | 53 | 56 | 58 | 16 |
| 20 R | 48 | 47 | 45 | 43 | 41 | 38 | 50 | 58 | 62 | 67 | 73 | 19 |
| 21 L | 44 | 41 | 39 | 36 | 34 | 31 | 49 | 53 | 57 | 62 | 65 | 18 |
| R | 41 | 38 | 35 | 31 | 29 | 27 | 46 | 48 | 51 | 55 | 60 | 18 |
| 22R | 46 | 43 | 40 | 36 | 33 | 30 | 47 | 50 | 53 | 55 | 58 | 18 |
| 23 L | 43 | 40 | 37 | 36 | 32 | 29 | 46 | 49 | 52 | 56 | 59 | 18 |
| R | 44 | 41 | 38 | 34 | 32 | 29 | 47 | 50 | 54 | 58 | 61 | 16 |
| 24 L | 45 | 41 | 39 | 36 | 36 | 34 | 48 | 52 | 56 | 63 | 67 | 17 |
| R | 40 | 37 | 35 | 33 | 32 | 28 | 44 | 49 | 53 | 56 | 59 | 20 |
| 25 L | 46 | 44 | 42 | 40 | 38 | 30 | 50 | 58 | 64 | 72 | 74 | 22 |
| R | 44 | 42 | 40 | 36 | 33 | 30 | 49 | 57 | 60 | 62 | 66 | 18 |
| Mean（°） | 43.44 ± 2.51 | 40.71±  2.80 | 38.05± 3.17 | 35.10± 3.22 | 32.51± 3.53 | 29.68± 3.64 | 45.90± 2.68 | 49.20± 4.01 | 52.10± 4.55 | 55.46± 5.51 | 58.17±  5.95 | 18.00±  1.64 |

**Acetabular cup’s anteversion at 11 positions**

| Hip number | Anteversion（°） | | | | | | | | | | | Inclination（°） |
| --- | --- | --- | --- | --- | --- | --- | --- | --- | --- | --- | --- | --- |
|  | 0mm | -1 | -2 | -3 | -4 | -5 | +1 | +2 | +3 | +4 | +5 |  |
| 1 L | 19 | 17 | 13 | 10 | 6 | 4 | 24 | 30 | 37 | 40 | 43 | 47 |
| 2 R | 22 | 21 | 13 | 8 | 3 | 2 | 24 | 26 | 27 | 30 | 31 | 40 |
| 3 L | 18 | 14 | 7 | 4 | 3 | 1 | 22 | 26 | 28 | 34 | 41 | 40 |
| R | 18 | 11 | 10 | 4 | 2 | 1 | 21 | 27 | 31 | 32 | 40 | 41 |
| 4 R | 14 | 12 | 10 | 9 | 8 | 4 | 18 | 19 | 25 | 30 | 36 | 40 |
| 5 L | 19 | 15 | 8 | 6 | 5 | 2 | 25 | 29 | 35 | 36 | 38 | 42 |
| R | 18 | 15 | 13 | 9 | 4 | 2 | 18 | 22 | 26 | 39 | 40 | 42 |
| 6 L | 17 | 10 | 9 | 8 | 5 | 4 | 19 | 26 | 29 | 38 | 43 | 41 |
| 7 L | 17 | 16 | 14 | 8 | 4 | 3 | 18 | 21 | 27 | 28 | 32 | 41 |
| R | 16 | 14 | 10 | 8 | 6 | 5 | 19 | 21 | 23 | 26 | 33 | 39 |
| 8 L | 16 | 12 | 10 | 8 | 6 | 4 | 19 | 26 | 30 | 32 | 39 | 41 |
| R | 17 | 15 | 11 | 6 | 4 | -6 | 17 | 24 | 28 | 34 | 43 | 45 |
| 9 L | 16 | 13 | 5 | 1 | -5 | -6 | 19 | 24 | 25 | 30 | 33 | 45 |
| R | 16 | 11 | 10 | 5 | 4 | 1 | 24 | 26 | 31 | 35 | 37 | 43 |
| 10 L | 17 | 15 | 14 | 12 | 9 | 2 | 21 | 24 | 28 | 36 | 46 | 47 |
| R | 18 | 13 | 6 | 5 | 3 | 1 | 20 | 23 | 34 | 38 | 45 | 46 |
| 11 L | 20 | 18 | 15 | 13 | 12 | 10 | 22 | 25 | 32 | 35 | 45 | 44 |
| R | 18 | 16 | 8 | 6 | 3 | -5 | 22 | 32 | 38 | 42 | 48 | 41 |
| 12 L | 18 | 13 | 9 | 2 | -2 | -5 | 21 | 25 | 27 | 33 | 37 | 44 |
| R | 19 | 13 | 8 | 2 | -2 | -6 | 24 | 28 | 33 | 38 | 44 | 41 |
| 13 L | 19 | 15 | 5 | 1 | -7 | -10 | 25 | 36 | 42 | 47 | 49 | 45 |
| 14 L | 18 | 11 | 3 | -3 | -8 | -12 | 24 | 28 | 36 | 43 | 48 | 43 |
| R | 20 | 16 | 10 | 9 | 6 | 1 | 21 | 23 | 26 | 33 | 38 | 46 |
| 15 L | 20 | 16 | 11 | 6 | 2 | -3 | 24 | 32 | 39 | 43 | 50 | 43 |
| 16 L | 20 | 14 | 10 | 4 | -5 | -12 | 24 | 33 | 40 | 45 | 54 | 46 |
| 17 L | 17 | 12 | 4 | -4 | -15 | -19 | 23 | 30 | 38 | 45 | 51 | 41 |
| R | 17 | 10 | 6 | 2 | -8 | -14 | 22 | 27 | 34 | 43 | 50 | 47 |
| 18 L | 19 | 15 | 9 | 6 | 3 | -9 | 23 | 30 | 40 | 47 | 56 | 48 |
| R | 18 | 15 | 8 | 5 | 2 | -5 | 25 | 31 | 38 | 46 | 50 | 42 |
| 19 L | 18 | 14 | 9 | 1 | -4 | -7 | 24 | 37 | 43 | 48 | 53 | 43 |
| R | 16 | 9 | 4 | 1 | -5 | -8 | 20 | 29 | 38 | 43 | 48 | 46 |
| 20 R | 19 | 17 | 13 | 10 | 5 | 4 | 25 | 33 | 37 | 42 | 51 | 48 |
| 21 L | 18 | 9 | -9 | -16 | -28 | -37 | 25 | 35 | 46 | 54 | 59 | 44 |
| R | 18 | 12 | 7 | -3 | -8 | -12 | 24 | 31 | 37 | 45 | 55 | 41 |
| 22R | 18 | 12 | 3 | -4 | -11 | -16 | 21 | 30 | 38 | 50 | 60 | 46 |
| 23 L | 18 | 12 | 8 | 5 | 4 | 3 | 20 | 26 | 30 | 36 | 39 | 43 |
| R | 16 | 9 | 4 | 2 | -3 | -6 | 17 | 20 | 26 | 33 | 39 | 44 |
| 24 L | 17 | 7 | 3 | -1 | -4 | -8 | 21 | 32 | 46 | 52 | 61 | 45 |
| R | 20 | 10 | 4 | -4 | -8 | -10 | 25 | 33 | 42 | 51 | 56 | 40 |
| 25 L | 22 | 17 | 16 | 13 | 11 | 5 | 25 | 37 | 51 | 56 | 71 | 46 |
| R | 18 | 11 | 6 | 1 | -7 | -10 | 22 | 27 | 36 | 44 | 47 | 44 |
| Mean（°） | 18.00±  1.64 | 13.34±  2.89 | 8.02± 4.72 | 4.02± 5.60 | -0.24± 7.70 | -4.07± 8.64 | 21.87± 2.51 | 27.90± 4.62 | 34.07± 6.72 | 38.80± 7.49 | 45.53±  8.88 | 43.44 ±2.51 |
